# Identifying Patients With Hypoglycemia Using Natural Language Processing: Systematic Literature Review

**DOI:** 10.2196/34681

**Published:** 2022-05-16

**Authors:** Yaguang Zheng, Victoria Vaughan Dickson, Saul Blecker, Jason M Ng, Brynne Campbell Rice, Gail D’Eramo Melkus, Liat Shenkar, Marie Claire R Mortejo, Stephen B Johnson

**Affiliations:** 1 Rory Meyers College of Nursing New York University New York, NY United States; 2 Department of Population Health Grossman School of Medicine New York University New York, NY United States; 3 Division of Endocrinology and Metabolism Department of Medicine University of Pittsburgh Pittsburgh, PA United States; 4 Division of Libraries New York University New York, NY United States; 5 Lehigh Valley Health Network Lehigh Valley Reilly Children’s Hospital Allentown, PA United States

**Keywords:** hypoglycemia, natural language processing, electronic health records, diabetes

## Abstract

**Background:**

Accurately identifying patients with hypoglycemia is key to preventing adverse events and mortality. Natural language processing (NLP), a form of artificial intelligence, uses computational algorithms to extract information from text data. NLP is a scalable, efficient, and quick method to extract hypoglycemia-related information when using electronic health record data sources from a large population.

**Objective:**

The objective of this systematic review was to synthesize the literature on the application of NLP to extract hypoglycemia from electronic health record clinical notes.

**Methods:**

Literature searches were conducted electronically in PubMed, Web of Science Core Collection, CINAHL (EBSCO), PsycINFO (Ovid), IEEE Xplore, Google Scholar, and ACL Anthology. Keywords included *hypoglycemia*, *low blood glucose*, *NLP*, and *machine learning*. Inclusion criteria included studies that applied NLP to identify hypoglycemia, reported the outcomes related to hypoglycemia, and were published in English as full papers.

**Results:**

This review (n=8 studies) revealed heterogeneity of the reported results related to hypoglycemia. Of the 8 included studies, 4 (50%) reported that the prevalence rate of any level of hypoglycemia was 3.4% to 46.2%. The use of NLP to analyze clinical notes improved the capture of undocumented or missed hypoglycemic events using International Classification of Diseases, Ninth Revision (ICD-9), and International Classification of Diseases, Tenth Revision (ICD-10), and laboratory testing. The combination of NLP and ICD-9 or ICD-10 codes significantly increased the identification of hypoglycemic events compared with individual methods; for example, the prevalence rates of hypoglycemia were 12.4% for International Classification of Diseases codes, 25.1% for an NLP algorithm, and 32.2% for combined algorithms. All the reviewed studies applied rule-based NLP algorithms to identify hypoglycemia.

**Conclusions:**

The findings provided evidence that the application of NLP to analyze clinical notes improved the capture of hypoglycemic events, particularly when combined with the ICD-9 or ICD-10 codes and laboratory testing.

## Introduction

### Background

Approximately 34 million (13%) US adults have diabetes [1]. Worldwide, 387 million persons have diabetes, a number that is expected to rise to 592 million by 2035 [2]. In 2017, direct and indirect costs attributed to diabetes in the United States were estimated to be US $327 billion [3]. Optimal glycemic control (glycated hemoglobin [HbA_1c_] <7%) can be achieved with comprehensive antidiabetic treatment; however, the risk of hypoglycemia increases. In patients with type 2 diabetes (T2D), after experiencing hypoglycemia, the 3-year incidence of cardiovascular events was 35.1%, and mortality 28.3% to 31.9% [4,5].

The incidence of hypoglycemia has been reported to vary widely for patients with diabetes. An earlier systematic review and meta-analysis of 46 studies found that 45% of the patients with T2D had mild or moderate hypoglycemia and 6% had severe hypoglycemia; the prevalence was even higher among those treated with insulin, with 50% having mild or moderate hypoglycemia events and 21% having severe events [6]. A subsequent review study showed that the rates of severe hypoglycemia in T2D were between 0.7 and 12 per 100 person-years in randomized controlled trials and between 0.2 (without treatment with insulin or sulfonylureas) and 2 (with treatment with insulin or sulfonylureas) per 100 person-years [7]. The most recent systematic review and meta-analysis of 72 studies indicated that the incidence rate of hypoglycemia was 14.5 to 42,890 episodes per 1000 person-years in type 1 diabetes (T1D) and 0.072 to 16,360 episodes per 1000 person-years in T2D [8].

The reported rates of hypoglycemia vary largely because of the marked heterogeneity in the way that hypoglycemia is defined, measured, and reported. Accurately identifying patients with hypoglycemia is key to preventing adverse events and mortality. There are several methods to identify hypoglycemia events and severity in large populations, including patient questionnaires and International Classification of Diseases, Ninth Revision (ICD-9), or International Classification of Diseases, Tenth Revision (ICD-10), and electronic health records (EHRs). Studies have found that using questionnaires [9] or International Classification of Diseases (ICD) codes [10] is often insensitive, leads to underestimation of hypoglycemia events, and is nonspecific in detecting hypoglycemia events.

EHRs have been widely adopted by health care systems, resulting in large amounts of data, including unstructured text in clinical notes [11,12]. The amount of unstructured text is vast and continues to grow at a breakneck pace. Clinical notes enable health care providers to not only identify patients at risk of hypoglycemia but also to obtain details on hypoglycemia; for example, symptomatic or asymptomatic hypoglycemia [13]. Once the patients at risk of hypoglycemia are identified, their treatment can be personalized, which helps to prevent future hypoglycemia and the resulting serious adverse effects. Traditional methods such as manual chart review can extract information related to hypoglycemia from EHR clinical notes [14]; however, such methods are time-consuming, labor intensive, and not scalable, which makes them impractical for use in large populations [15].

By contrast, novel data science approaches, including using natural language processing (NLP), have been applied to overcome the aforementioned difficulties [16]. NLP, a form of artificial intelligence, uses computational algorithms to process human language content for a variety of purposes [17]. The application of NLP algorithms is a scalable, efficient, and quick method to extract unstructured data from a large population [18,19]. Applications of NLP in the health domain can be categorized into 2 groups: rule-based methods and machine learning methods [20]. Rule-based NLP techniques are based on a predefined clinical vocabulary, which identifies a set of core concepts for target extraction (eg, hypoglycemia), and may also use pattern matching (such as regular expressions) and filters [21,22]. Rule-based systems are time-consuming to set up, but they are easy to understand and modify and often require fewer amounts of data than machine learning approaches [21,23,24]. Machine learning systems leverage the same feature sets as those used in rule-based systems but do the work to discover the rules needed for a solution; however, this comes at a price: the resulting systems often function as a *black box*, which is difficult for humans to understand and trust [20]. In addition, machine learning systems typically require very large sample sizes for development [23]. Deep learning approaches (neural networks) are a form of machine learning used in recent years [25,26], which can achieve performances comparable with, or better than, those of domain experts in identifying clinical information [16]. However, deep learning–based models require large amounts of training data to achieve high accuracy, hindering the adoption of deep learning–based models in scenarios with limited amounts of training data [27]. As a result, state-of-the-art deep learning methods of NLP (eg, transformer models and transfer learning) were developed to address these issues, and they have been proven to be extremely effective in the NLP domain [27,28].

### Objectives

Currently, little is known about what types of NLP algorithms were applied to identify hypoglycemia and how differences in hypoglycemia incidence identified from unstructured data using NLP compare with hypoglycemia incidence identified from structured data (eg, ICD codes) across studies. It was reported in 1 study that a higher number of hypoglycemia events could be identified in clinical notes by using NLP than by using ICD codes (65% vs 20%, respectively) [29]. Thus, in this systematic review, we aimed to synthesize the literature on the application of NLP to extract hypoglycemia from EHR clinical notes and compare the differences between hypoglycemia incidence identified from unstructured data using NLP and hypoglycemia incidence identified from structured data (eg, ICD codes) across studies.

## Methods

### Search Strategies

Literature searches for a comprehensive review were conducted in 7 electronic databases: PubMed, Web of Science Core Collection, CINAHL (EBSCO), PsycINFO (Ovid), IEEE Xplore, Google Scholar, and ACL Anthology. The search strategies were developed in consultation with a health sciences librarian (BCR). The searches were conducted before February 22, 2022. Database-specific subject headings (eg, Medical Subject Headings) and relevant keywords were identified to describe hypoglycemia and these terms were searched in combination with terms related to NLP. As few articles related to hypoglycemia and NLP were located, the searches were widened to include broader terms such as *blood sugar* or *blood glucose*, and *machine learning* or *artificial intelligence*. No date, language, or publication filters were applied within the databases. Appropriate Boolean operators were used to structure the search queries and both unqualified free-text searching and field tags were used; the detailed search queries for each database are presented in Textbox 1.

**Search strategies for hypoglycemia and natural language processing.**

**PubMed**

- ((*Hypoglycemia* [MeSH]) OR *Blood Glucose* [MeSH] OR hypoglycemi* [TW] OR blood sugar* [TW] OR *blood glucose* [TW]) AND ((((Natural Language Processing [MeSH]) OR *Machine Learning* [MeSH]) OR *Artificial Intelligence* [MeSH]) OR *Data Mining* [MeSH:noexp] OR *NLP* [TW] OR *natural language processing* [TW] OR *machine learning* [TW] OR *artificial intelligence* [TW] OR *text mine* [TW] OR text analys* [TW] OR *text processing* [TW] OR text classif* [TW] OR *information extraction* [TW] OR ((electronic health record* [TW]) AND (diagnos* [TW])))

**Web of Science**

- TS=(hypoglycemi* OR (*blood NEAR/3 sugar*) OR (*blood NEAR/3 glucose*)) AND TS=((*“Natural Language Processing”* OR *NLP* OR *“Machine Learning”* OR *“Artificial Intelligence”* OR (*“text mining”* OR *“text mine”* OR “text analys*” OR “text* analyst” OR “text* processing” OR “text classif*” OR *“information extraction”)* OR ((electronic health record* OR electronic medical record* OR electronic patient record*) AND diagnos*)))
- Document type: article
- Language: English

**CINAHL**

- ((MH “*Hypoglycemia*”) OR (MH “*Blood Glucose”*) OR (hypoglycemi* OR *“blood glucose”* OR *“blood sugar”*)) AND (( (MH “*Natural Language Processing*”) OR *“natural language processing”* OR (MH “*Artificial Intelligence*+”) OR (MH “*Data Mining”*) OR (MH “*Machine Learning*+”) OR “*text mining”* OR “*text analysis”* OR “*text processing”* OR text classif* OR “*information extraction”* ) OR ( ((“*electronic health record”* OR “*electronic medical record”* OR “*electronic patient record”* OR “*electronic health records”* OR “*electronic medical records*” OR “*electronic patient records”* OR *EHR* OR *EMR*) N3 diagnos*) ))

- Language: English

**PsycINFO (Ovid)**

- (hypoglycemi*.mp. or *exp Hypoglycemia/ or blood sugar.mp.* or *exp Blood Sugar/ or blood glucose.mp.)* AND *(natural language processing.mp.* or *exp Natural Language Processing/* or *machine learning.mp.* or *exp Machine Learning/* or *artificial intelligence.mp.* or *exp Artificial Intelligence/* or *text mining.mp.* or *text processing.mp.* or text classif*.mp. or *information extraction.mp.* or *((exp Electronic Health Records/* or *electronic health record.mp.* or *electronic medical record.mp.* or *electronic patient record.mp.)* and *(exp Diagnosis/* or diagnos*.mp.)))

**ACL Anthology**

- *hypoglycemia* OR *blood glucose* OR *blood sugar* OR *hypoglycemic*

**Google Scholar**

- *natural language processing* AND *hypoglycemia* AND *electronic health records*

**IEEE Xplore**

- (*All Metadata:blood sugar* OR *All Metadata:blood glucose* OR *All Metadata:hypoglycemia* OR *All Metadata:hypoglycemic)* AND (*All Metadata:natural language processing* OR *All Metadata:NLP* OR *All Metadata:“machine learning”* OR *All Metadata:“artificial intelligence”* OR *All Metadata:“text mining”* OR *All Metadata:“text analysis”* OR *All Metadata:“text analyses”* OR *All Metadata:“text analytics”* OR *All Metadata:“text processing”*)
- Filters applied: journals

### Inclusion and Exclusion Criteria

The inclusion criteria were as follows: studies that (1) were restricted to participants aged ≥18 years; (2) reported a sample with a diagnosis of diabetes; (3) applied NLP to identify hypoglycemia; (4) reported the number or percentage of participants who had experienced at least one hypoglycemic episode, the incidence of hypoglycemic episodes experienced, or data to allow the calculation of one of these measures; (5) used EHR data; (6) were published as full papers in peer-reviewed journals; (7) were published in English. No restrictions were applied regarding the definition or measurement of hypoglycemia. No restrictions were applied to country or origin of the studies. Studies were excluded if (1) they did not report outcomes related to hypoglycemia, (2) they were pharmacological trials or the intervention focused on treatment or care, (3) the participants were all pregnant or children, and (4) they reported only conference papers or proceedings.

### Data Extraction

**Figure 1:**
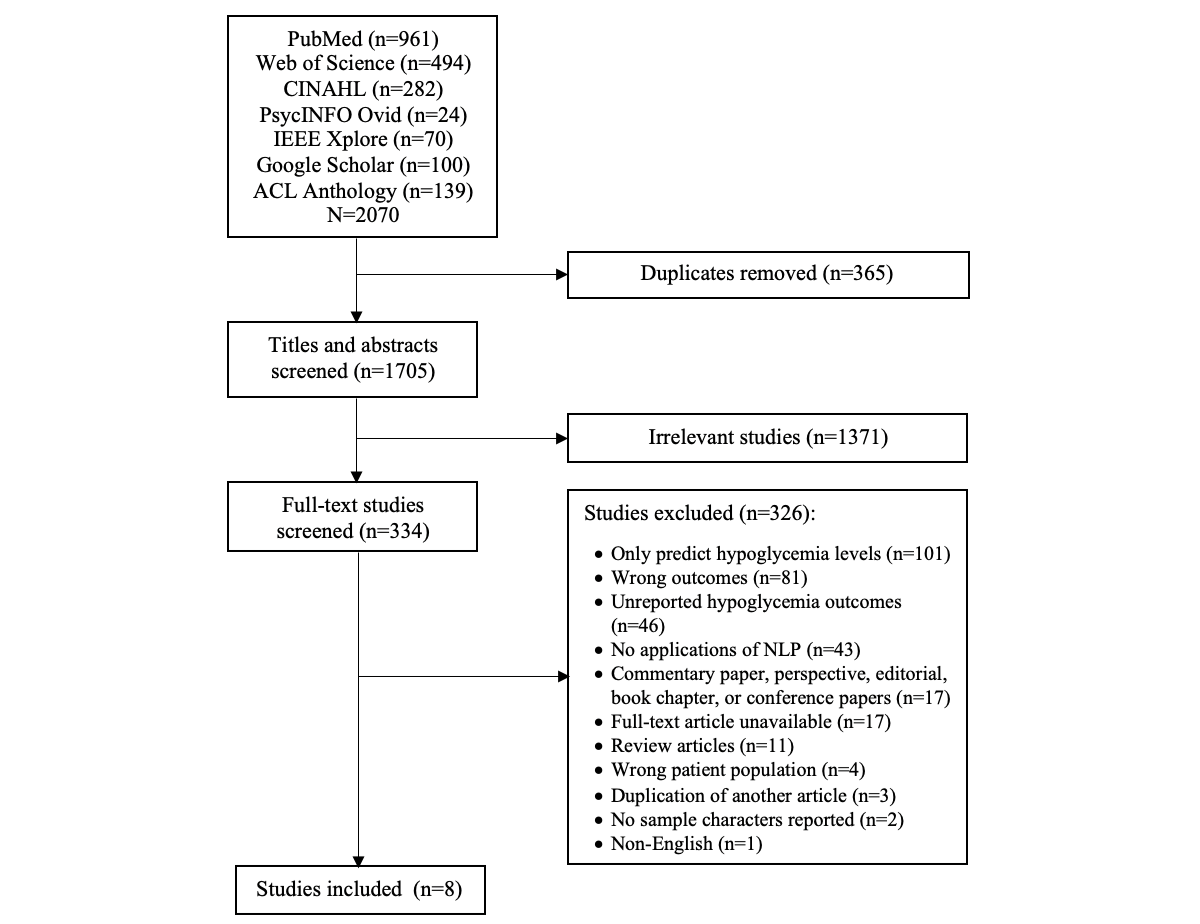
PRISMA (Preferred Reporting Items for Systematic Reviews and Meta-Analyses) flowchart. In the case of Google Scholar, the first 100 results based on relevancy ranking is suggested to identify additional articles, and in the case of ACL Anthology, all the citations found were added to the irrelevant set (excluded based on title and abstract) [30]. NLP: natural language processing.

We first developed and tested a data extraction form, with adaptations made accordingly. The titles, abstracts, and full-text articles were screened by 2 independent reviewers (MCRM, LS, Emily M Pan, or Yi Lan Zhang). Once conflicts were identified, agreement was reached after discussion with the third reviewer (YZ). The results related to the identification of eligible studies were summarized according to the PRISMA (Preferred Reporting Items for Systematic Reviews and Meta-Analyses) guidelines (Figure 1). The searches yielded 2070 citations, and after removing duplicates, 1705 (82.37%) titles and abstracts were screened for eligibility. After full-text retrieval of 334 potentially relevant papers, 326 (97.6%) were subsequently excluded, leaving 8 (2.4%) papers that applied NLP to identify hypoglycemia and reported the rates of hypoglycemia that were eligible for inclusion in the analyses. The reference sections of the relevant articles were searched manually, but no further relevant articles were found. Studies were summarized based on the following categories: authors and country, sample size and characteristics, medical conditions, antihyperglycemic medication, study design, data source, definition of hypoglycemia, method used to identify hypoglycemia, NLP algorithm (eg, rule-based or machine learning), NLP algorithm validation, and outcomes (Tables 1 and 2).

**Table 1:** Summary of studies on natural language processing (NLP) and hypoglycemia.

Author, year, country	Sample characteristics	Medical conditions	Antihyperglycemic medication	Study design
Nunes et al, 2016 [31], United States	N=844,683; age (years; n [%]): <30: 10,138 (1.20), 30 to 39: 38,491 (4.56), 40 to 49: 105,476 (12.49), 50 to 59: 196,494 (23.26), 60 to 69: 232,885 (27.57), >69: 261,199 (30.92); female (n [%]): 433,322 (51.30); White (n [%]): 655,474 (77.60); T2D^a^ (n [%]): 844,683 (100); baseline measures, mean (SD): BMI (kg/m^2^): 31.8 (10.2), HbA_1c_^b^ (%): 7.0 (1.9), blood glucose level (mg/dL): 139.0 (82)	Atrial fibrillation (n [%]): 60,773 (7.19); hypertension (n [%]): 555,482 (65.76); hyperlipidemia (n [%]): 510,944 (60.49); cerebrovascular disease (n [%]): 54,336 (6.43); chronic kidney disease: retinopathy (n [%]): 10,356 (1.23), neuropathy (n [%]): 44,352 (5.25), nephropathy (n [%]): 26,498 (3.14); ischemic heart disease (n [%]): 154,049 (18.24); congestive heart failure (n [%]): 59,438 (7.04)	Not specified	Retrospective cohort study
Nunes et al 2017 [29], United States	N=143,635; age (years; n [%]): <30: 1333 (0.93), 30 to 39: 5420 (3.77), 40 to 49: 15,645 (10.89), 50 to 59: 32,796 (22.83), 60 to 69: 39,852 (27.75), >69: 48,491 (33.76); female (n [%]): 69,879 (48.65); White (n [%]): 116,701 (81.25); T2D (n [%]): 143,635 (100); baseline measures (median [IQR]): BMI (kg/m^2^): 32.3 (28.1-37.6), HbA_1c_ (%): 7.1 (6.5-8.1), blood glucose level (mg/dL): 146.0 (116.0-191.0)	N=143,635; cerebrovascular disease (n [%]): 11,903 (8.29); retinopathy (n [%]): 3091 (2.15); neuropathy (n [%]): 12,961 (9.02); nephropathy (n [%]): 8338 (5.80); ischemic heart disease (n [%]): 33,570 (23.37)	N=143,635; sulfonylureas (n [%]): 143,635 (100)	Retrospective cohort study
Loughlin et al, 2018 [32], United States	N=6024; EQW^c^ cohort (n [%]): 2008 (33.33%); age (years): —^d^; female (n [%]): 1004 (50); White (n [%]): 1630 (81.17); T2D (n [%]): 2008 (100); baseline measures: —; BI^e^ cohort (n [%]): 4016 (66.67%); age (years): —; female (n [%]): 2036 (50.70); White (n [%]): 3277 (81.60); T2D (n [%]): 4016 (100); baseline measures: —	—	N=6024; insulin (n [%]): 6024 (100)	Retrospective cohort study
Pettus et al, 2019 [33], United States	N=831,456; BI switchers (n=3920 to 19,256); age (years): range 58.2-60.1; female (%): range 49.8-52.0; White (%): —; T2D: (831,456, 100%); baseline measures: BMI (kg/m^2^): range 33.8-35.0; HbA_1c_ (%): range 8.91-9.02; blood glucose level (mg/dL): —; smoking (%): —. Insulin naïve (n=2279 to 47,085); age (years): range 58.8-60.4; female (%): range 48.6-52.1; White (%): —; T2D (n [%]): (100); baseline measures: BMI (kg/m^2^): range 34.0-34.6; HbA_1c_ (%): range 9.39-9.64; blood glucose level (mg/dL): —; smoking (%): —	BI switchers: hypertension: 63.4-73.4, hyperlipidemia: 68.1-77.8, microvascular complication: 44.7-55.7, macrovascular complication: 44.2-63.5. Insulin naïve: hypertension: 56.8-74.2; hyperlipidemia: 61.5-77.8, microvascular complication: 25.3-34.6, macrovascular complication: 32.7-63.5	BI switchers: sulfonylureas: 24.5-28.3; any OAD^f^: 63.6- 75.2. Insulin naïve: sulfonylureas: 47.6-56.6;	Retrospective cohort study
Li et al, 2019 [34], United States	N=38,780; age (years), mean: 57.0; female (n [%]): 21,716 (56); White (%): 18,226 (47); T2D (%): —; baseline measures: BMI (kg/m^2^), mean (SD): 35.7 (9.8); HbA_1c_ (n [%]): ≤6.5%: 5321 (13.72), >6.5% to <7%: 1840 (4.74), ≥7% to <8%: 3155 (8.14), ≥8% to <9%: 1773 (4.57), ≥9%: 3977 (10.26), missing: 22,714 (58.57)	N=38,780; coronary artery disease (n [%]): 2021 (5.21); chronic heart failure (n [%]): 1582 (4.08); diabetic neuropathy (n [%]): 1414 (3.65)	N=38,780; long-acting insulin (LAI^g^): 615 (1.59); sulfonylureas: 8727 (22.50)	Retrospective cohort study
Misra-Hebert et al, 2020 [35], United States	N=204,517; the values provided herein are from a subsample: (n=46,302); age (years): 61.48; female (%):22,633 (48.90); White (%):34,004 (73.40); T2D (n [%]):46,302 (100); baseline measures: BMI (kg/m^2^): 32.2; HbA_1c_ (%): 6.6; blood glucose level (mg/dL): —	n=46,302; cardiovascular disease (n [%]): 13,372 (28.9); congestive heart failure (n [%]): 2195 (4.7); chronic kidney disease (n [%]): 2460 (5.3)	n=46,302; insulin (n [%]): 8050 (17.4); glucagon-like peptide-1 receptor agonist (n [%]): 1781 (3.8); dipeptidyl peptidase 4: 4437 (9.6); sodium-glucose cotransporter-2 inhibitor (n [%]): 791 (1.7); metformin: 28,851 (62.3); sulfonylureas (n [%]): 10,098 (21.8); alpha-glucosidase inhibitor (n [%]): 107 (0.2)	Retrospective cohort study
Uzoigw et al 2020 [36], United States	N=359,087; T2D (n [%]): 317,399 (88.39); age (years), median (IQR): 68.0 (18); female (n [%]): 154,512 (48.68); White (%):121,468 (38.27); baseline measures: BMI (kg/m^2^): —; HbA_1c_ (%): —; blood glucose level (mg/dL): —; smoking (n [%]):106,760 (33.63). T1D^h^: (n [%]): 41,688 (11.61); age (years): median (IQR) 55.0 (30); female (n [%]): 21,034 (50.46); White (n [%]): 16,072 (38.55); baseline measures: BMI (kg/m^2^): —; HbA_1c_ (%): —; blood glucose level (mg/dL): —; smoking (n [%]): 9174 (22)	T2D: N=317,399; hypertension (n [%]): 257,093 (81); hyperlipidemia (n [%]): 193,616 (61); cardiovascular disease (n [%]): 158,699 (50). T1D: N=41,688; high blood sugar level or diabetic ketoacidosis (n [%]): 14,067 (33.74); cancer (n [%]): 6752 (16.20); stroke (n [%]): 7377 (17.70); substance use or abuse (n [%]): 4917 (11.79)	T2D: N=317,399; insulin (n [%]): 174,569 (55); sulfonylureas (n [%]): 55,710 (17.55); metformin (n [%]): 114,263(36). T1D: N=41,688; insulin (n [%]): 37,279 (89.42); sulfonylureas (n [%]): 1846 (4.43); metformin (n [%]): 5059 (12.14)	Retrospective cohort study
Ganz et al 2014 [37], United States	N=7235; HbA_1c_ (%): —; blood glucose level (mg/dL): —; smoking (%): —; T2D (n [%]): 7235 (100); age (years), mean (SD): 60.82 (11.65); female (n [%]): 3668 (50.70); White (n [%]): 4576 (63.25); baseline measures: BMI (kg/m^2^): —; HbA_1c_ (%): —; blood glucose level (mg/dL): —; smoking (%): —	T2D (n [%]): 7235 (100)	Insulin: glargine (%): 77.24; neutral protamine Hagedorn insulin (%): 5.86; detemir (%): 16.90. Sulfonylureas (%): 38.06; metformin (%): 36.66; other OADs (%): 25.82	Retrospective cohort study

^a^T2D: type 2 diabetes.

^b^HbA_1c_: glycated hemoglobin.

^c^EQW: exenatide once weekly.

^d^Not available.

^e^BI: basal insulin.

^f^OAD: oral antidiabetic drug.

^g^LAI: long-acting insulin.

^h^T1D: type 1 diabetes.

**Table 2:** Summary of studies on natural language processing (NLP) and hypoglycemia.

Author, year, country	Data source	Definition of hypoglycemia	Method used to identify hypoglycemia	NLP algorithm: rule-based or machine learning	NLP algorithm validation	Outcomes
Nunes et al, 2016 [31], United States	Optum Humedica EHR^a^ database, which incorporates EHRs from 35 large medical provider organizations (including >195 hospitals), >25,000 physicians, and >25 million patients, making up the largest EHR database within the United States (January 2009 to March 2014)	Serious: ICD-9^b^ identified events were characterized as serious or nonserious if the diagnosis was identified within a problem list; NLP-identified categories included serious (eg, serious, acute, severe, and profound); mild to moderate: NLP-identified categories included mild to moderate (eg, mild, moderate, slight, and minor)	ICD-9 algorithm (structured diagnostic codes only); NLP algorithm (NLP of clinical notes); combined algorithm (either ICD-9 diagnostic codes or NLP of clinical notes)	Rule-based	The final algorithm was validated by manual review: precision (PPV^c^)=0.77, recall (sensitivity)=0.67	Period prevalence (%): any conditions: ICD-9: 12.37, NLP: 25.11, combined: 32.19; serious: ICD-9: 11.93, NLP: 10.71, combined: 18.72; mild to moderate: ICD-9: 0.00, NLP: 0.76, combined: 0.78. Incidence rate (per 100 person-years): any conditions: ICD-9: 2.25, NLP: 4.78, combined: 6.28. Serious: ICD-9: 2.12, NLP: 1.72, combined: 3.19; mild to moderate: ICD-9: 0.00, NLP: 0.09, combined: 0.08. Event rate (per 100 person-years): any conditions: ICD-9: 6.92, NLP: 10.03, combined: 16.12; serious: ICD-9: 6.63, NLP: 3.06, combined: 8.90; mild to moderate: ICD-9: 0.00, NLP: 0.20, combined: 0.19
Nunes et al, 2017 [29], United States	Optum EHR database (January 2009 to December 2014)	Serious: ICD^d^ and CPT^e^ evidence of medical intervention or abstracted descriptors suggestive of serious event; nonserious, mild to moderate: No ICD or CPT evidence of medical intervention but with abstracted descriptors suggestive of mild to moderate event; nonserious, unspecified: no ICD or CPT evidence of medical intervention and no descriptors of event seriousness	ICD codes and NLP	Rule-based	The final algorithm was validated by manual review: precision (PPV)=0.77, recall (sensitivity)=0.67	Incidence rate (per 100 person-years; 95% CI): any conditions: overall: 11.76 (11.49-12.04), sulfonylureas use: 12.77 (12.40-13.15), sulfonylureas nonuse: 10.39 (10.00-10.79). Serious: overall: 5.06 (4.88-5.24), sulfonylureas use: 5.77 (5.52-6.03), sulfonylureas nonuse: 4.09 (3.84-4.34). Nonserious, mild to moderate: overall: 0.14 (0.11-0.17), sulfonylureas use: 0.17 (0.13-0.22), sulfonylureas nonuse: 0.09 (0.06-0.13). Nonserious, unspecified: overall: 6.57 (6.37-6.78), sulfonylureas use: 6.83 (6.56-7.11), sulfonylureas nonuse: 6.21 (5.91-6.52)
Loughlin et al, 2018 [32], United States	Optum EHR database (January 2012 to January 2015)	Documented blood glucose level <3.9 mmol/L or emergency physician–charted diagnosis of hypoglycemia	Hypoglycemia and gastrointestinal symptoms (vomiting, nausea, diarrhea, or constipation) were identified by using both ICD-9 Clinical Modification diagnostic codes within structured fields and NLP clinical notes; hypoglycemia was identified using an algorithm developed by Optum, incorporated diagnostic codes, and NLP of clinical notes	Rule-based	The final algorithm was validated by manual review: precision (PPV)=0.77, recall (sensitivity)=0.67	Incidence rate (per 1000 person-years; 95% CI): EQW^f^ cohort: 52.5 (44.4-61.6), BI^g^ cohort: 65.7 (59.1-72.7). Any gastrointestinal symptoms: EQW cohort: 225.5 (206.8-245.5), BI cohort: 191.0 (179.1-203.6). Participants with at least one event (n/N [%]): EQW cohort: 149/2008 (7.42), BI cohort: 368/4016 (9.16). Any gastrointestinal symptoms (n/N [%]): EQW cohort: 534/2008 (26.60), BI cohort: 946/4016 (23.56)
Pettus et al, 2019 [33], United States	Optum Humedica EHR database (January 1, 2007, to March 31, 2017)	Hypoglycemia: ICD-9 and ICD-10^h^ codes for hypoglycemia; plasma glucose level measures ≤70 mg/dL; IM^i^ glucagon administration; NLP: mention of hypoglycemia; severe hypoglycemia: ICD-9 and ICD-10 codes for hypoglycemia that is severe by default or ICD-9 and ICD-10 codes for hypoglycemia and hypoglycemia is reason for care on discharge or admission or hypoglycemia index date on same day as emergency department visit or inpatient diagnosis on admission (all related to hypoglycemic coma); plasma glucose level measures <54 mg/dL; IM glucagon administration; NLP: mention of hypoglycemia with either a descriptor of hypoglycemia severity, including severity terms (eg, severe) and attributes (eg, emergency), or emergency department visit or inpatient admission on same day as medical record was written	ICD-9 and ICD-10 codes; plasma glucose measures ≤70 mg/dL; IM glucagon administration; NLP	Rule-based	The final algorithm was validated by manual review: precision (PPV)=0.77, recall (sensitivity)=0.67	Any hypoglycemia (%): BI switchers: 42.2-46.2. Insulin naïve: 22.8-28.8. Severe hypoglycemia: BI switchers: 8.2-17.4, insulin naïve: 2.7-8.6
Li et al, 2019 [34], United States	Regenstrief Medical Record System, which is an urban safety-net medical institution in Indianapolis, Indiana, United States. In 2012, Eskenazi Health had 1081 physicians on staff and serviced 950,592 outpatient visits, including 234,637 community health center visits (January 1, 2004, to December 31, 2013)	Plasma or point-of-care glucose value of at least 5 mg/dL and <70 mg/dL, documented in the medical record; ICD-9 code: 251.1 or 251.2; ICD code 250.8 without any of the following codes: 259.8, 272.7, 681.xx, 682.x, 686.9, 707.1x, 707.2x, 707.8, 707.9, 709.3, 730.0x, 730.1x, 730.2x, 731.8; text note indicating hypoglycemia, including a blood glucose value	Laboratory tests; diagnostic codes; NLP	Rule-based	—	A 1-year window for prior episodes of hypoglycemia: overall prevalence (n/N [%]): 8182/38,780 (21); non-LAI^j^ and sulfonylureas within 90 days (%): 42.92; sulfonylureas without insulin (%): 23.82; no insulin, no sulfonylureas (%): 17.85%; blood glucose value between 5 mg/dL and 70 mg/dL (n/N [%]): 7070/38,780 (18.23); blood glucose value<54 mg/dL (n/N [%]): 4784/38,780 (12.34); NLP (n/N [%]): 3751/38,780 (9.67), with 539/38,780 (1.39), identified only by NLP
Misra-Hebert et al, 2020 [35], United States	Cleveland Clinic Health System patient records (2005 to 2017)	Hypoglycemia: blood glucose level <70 mg/dL; severe hypoglycemia: patients with T2D^k^ require hospitalization or emergency department visit; nonsevere hypoglycemia: does not require assistance for recovery	NLP; ICD-9 codes: 251.0, 251.1, 251.2; ICD-10 codes: E08.641, E11.641, E11.649, E13.64, E13.641, E13.649, E16.0, E16.1, E15, E16.2	Rule-based	Compared with clinician chart review manually, PPV=93%	Prevalence: among 204,517 patients with no codes for nonsevere hypoglycemia, evidence of nonsevere hypoglycemia was found in 7035 (3.4%) using NLP. Number of nonsevere hypoglycemia events: ICD codes (n/N [%]): 10,205/204,517 (4.99), NLP: 14,763/204,517 (7.22), with overlap of only 5 events. Incidence proportion of patients from 2005 to 2017 ICD codes (%): severe hypoglycemia: 0.3 to 1.7, nonsevere hypoglycemia: 0.4 to 1.3; NLP+ICD (%): nonsevere hypoglycemia: 0.8 to 2.6
Uzoigw et al, 2020 [36], United States	Amplity Insights database, unstructured health records, generated from provider notes as transcribed from verbal to written form (January 1, 2016, to April 30, 2018)	Nonsymptom-based: mention of hypoglycemia, low blood glucose level or blood glucose value≤70 mg/dL; symptom-based: keywords identified by endocrinologists, used by patients to describe hypoglycemia	ICD codes; NLP	Rule-based	—	Prevalence during 2 years (%): T2D: ICD: 52 (<0.1); combined symptom and nonsymptom-based: 11.4; nonsymptom-based: 7.59; symptom-based: irritable or anxious: 14.50; cognitive issues: 12.14; elevated or irregular heart rate: 10.21. T1D^l^: ICD codes: 30 (0.1); combined symptom and nonsymptom-based: 20.4; nonsymptom-based: 18.12; symptom-based: irritable or anxious: 16.00; cognitive issues: 8.17; elevated or irregular heart rate: 8.17
Ganz et al, 2014 [37], United States	Humedica real-time longitudinal clinical data patient-level EHR database (January 2008 to December 2011)	Severe hypoglycemia: blood glucose level≤40 mg/dL	ICD-9 codes 251.0x, 251.1x, 251.2x, or 250.3x on different days; NLP	Rule-based	The final algorithm was validated by manual review: precision (PPV)=0.77, recall (sensitivity)=0.67	Posttitration follow-up period (1.8 years): incidence rate (per 100 patient-years; 95% CI)=4.63 (4.59-4.67); total severe hypoglycemia rate (per 100 patient-years)=9.69 (9.64-9.75). Incidence rate for patients with history of severe hypoglycemia events (95% CI)=5.91 (5.76-6.06). Total severe hypoglycemia rate for patients with history of severe hypoglycemia events (95% CI)=9.00 (8.87-9.12)

^a^EHR: electronic health record.

^b^ICD-9: International Classification of Diseases, Ninth Revision.

^c^PPV: positive predictive value.

^d^ICD: International Classification of Diseases.

^e^CPT: Current Procedures Terminology.

^f^EQW: exenatide once weekly.

^g^BI: basal insulin.

^h^ICD-10: International Classification of Diseases, Tenth Revision.

^i^IM: intramuscular.

^j^LAI: long-acting insulin.

^k^T2D: type 2 diabetes.

^l^T1D: type 1 diabetes.

## Results

### Description of Included Studies

All included studies (n=8) were conducted in the United States [29,31-37]. The sample sizes were large, ranging from 6024 to 844,683. Of the 8 studies, 6 (75%) included only T2D [29,31-33,35,37], 1 (13%) included both T1D and T2D [36], and 1 (13%) did not specify the type of diabetes [34]. The participants varied in age from 57 to 68 years, and 48.7% to 56% were women. Among the studies (7/8, 88%) that reported on ethnicity, the percentage of non-White participants ranged from 18.8% to 62%. Mean BMI ranged from 31.8 (SD 10.2) to 35.7 (SD 9.8) kg/m^2^, and mean HbA_1c_ ranged from 6.6% to 9.64%. Varied comorbidities were reported; for example, hypertension, hyperlipidemia, ischemic heart disease, and heart failure. Of the 8 studies, 4 (50%) provided diabetes-related complications, including retinopathy, neuropathy, and nephropathy [29,31,33,34]; 6 (75%) reported that 1.6% to 100% of the participants injected insulin [32-37]; and 6 (75%) reported 4.4% to 100% sulfonylureas use [29,33-37].

All the included studies (n=8) were retrospective cohort study designs, with the observational durations of the cohort ranging from 2 to 12 years. Population samples were obtained from varied EHR databases such as Optum Humedica [29,31-33,37], Regenstrief [34], Cleveland Clinic Health System patient records [35], and Amplity Insights [36].

### Methods of Identifying Hypoglycemia

All included studies used a combination of ICD codes and NLP to identify hypoglycemia; other methods were applied, including laboratory tests for plasma glucose measures ≤70 or <54 mg/dL [33,34] and glucagon administration [33]. ICD-9 and ICD-10 codes used to identify hypoglycemia were described in detail by Misra-Hebert et al [35]. Of the 8 studies, 3 (38%) reported both serious (level 3) and mild or moderate hypoglycemia (levels 1 and 2) [29,31,35], 1 (13%) reported both overall unspecified and severe hypoglycemia [33], and 3 (38%) reported data on unspecified hypoglycemia [32,34,36], whereas 2 (25%) studies also reported symptom-based and nonsymptom-based hypoglycemia [32,36].

### NLP Algorithms Applied to Identify Hypoglycemia

All included studies applied rule-based algorithms (Table 3). The study by Misra-Hebert et al [35] described in detail the NLP steps, including splitting clinical notes into sentences and phrases, filtering sentences and phrases to those containing references to a hypoglycemia-related Unified Medical Language System [38] concept, identifying temporal phrases (identifying when the event occurred), and clarifying polarity (assertion or negation) into no, nonsevere, or severe event using both rule-based algorithms. Li et al [34] identified hypoglycemia using a formally defined pattern (*regular expression*) [39] such as a *blood sugar word,* followed within 5 words by what could be a low blood sugar value represented by a number ranging from 10 to 69. Uzoigwe et al [36] identified keywords or concepts of interest related to both symptom-based and nonsymptom-based hypoglycemic events. The remaining studies (5/8, 63%) applied the same NLP algorithms to identify [29,31-33,37] (1) terms or concepts (eg, hypoglycemia), including alternative or incorrect spellings and abbreviations; (2) descriptive attributes of the hypoglycemia mention (eg, seriousness, duration, and frequency); (3) sentiment of the mention (eg, denial, affirmation, and discussion); and (4) other contextual information (eg, note section headers and neighboring text).

Manual review of clinical notes was used as the gold standard to validate the NLP algorithms in 63% (5/8) of the studies. Of the 8 studies, 2 (25%) did not report validation of the algorithm, whereas in the 6 (75%) reporting studies, the precision (positive predictive value) for the hypoglycemia algorithm was 0.77% to 93% [29,31-33,35,37]. Of these 6 studies, 5 (83%) reported that the recall (sensitivity) was 0.67 [29,31-33,37].

**Table 3:** Natural language processing (NLP) algorithms applied in the reviewed studies.

Study	NLP algorithm type	Details of NLP algorithms
Ganz et al, 2014 [37]; Nunes et al, 2016 [31]; Nunes et al, 2017 [29]; Loughlin et al, 2018 [32]; Pettus et al, 2019 [33]	Rule-based	Identify terms consistent with hypoglycemia (including alternative or incorrect spellings and abbreviations) Identify descriptive attributes of the hypoglycemia mention (eg, seriousness, duration, and frequency) Identify sentiment of the mention (eg, denial and affirmation, including “has,” “diagnosed,” and “present”) Identify contextual information (eg, note section headers and neighboring text). Sections such as “history of present illness,” “assessment,” “hospital course,” “reason,” “review of symptoms,” and “chief complaint” generally reflected occurrence of hypoglycemia
Li et al, 2019 [34]	Rule-based	A formally defined pattern (regular expression), which identified clinical reports mentioning a “blood sugar word” followed within 5 words by what could be a low blood sugar value represented by a number ranging from 10 to 69
Misra-Hebert et al, 2020 [35]	Rule-based	Split clinical notes into sentences and phrases Filter sentences and phrases to those containing a hypoglycemia-related Unified Medical Language System concept Identify temporal phrases (when the event occurred) Classify polarity (assertion or negation) into no, nonsevere, and severe event
Uzoigwe et al, 2020 [36]	Rule-based	Identify keywords or concepts of interest: symptom-based and nonsymptom-based hypoglycemic events Symptom-based terms: neuroglycopenic and adrenergic symptomology associated with hypoglycemia. Adrenergic symptomology: elevated or irregular heart rate, sweating, tremor, trembling, tingling, or shaking, and vision impairment Neuroglycopenic symptomology: cognitive issues, irritable or anxious, mood or behavior change+NOT substance abuse or alcohol, slurred speech+NOT stroke+NOT substance abuse or alcohol Nonsymptom-based definition: Mention of “hypoglycemia” Relevant medical ontology such as “low glucose” A blood glucose laboratory value ≤70 mg/dL documented

### Prevalence or Incidence of Hypoglycemia

The prevalence or the incidence of hypoglycemia largely varied across studies. All studies used a combination of NLP and other approaches (eg, ICD codes) to identify hypoglycemia. Overall, the prevalence rate of any condition of hypoglycemia was 3.4% to 46.2%, as reported by 50% (4/8) of the studies [31,33,34,36], and the incidence rate was 6.28% to 65.7%, as reported by 38% (3/8) of the studies [29,31,32]. The prevalence rate of nonsevere hypoglycemia was 0.1% to 3.4% [29,31,35] and that of severe hypoglycemia was 5.1% to 18.7% [29,31,33,37]. Of the 8 studies, 4 (50%) compared the prevalence or incidence of hypoglycemia identified by NLP and ICD codes. In the study by Nunes et al (2016) [31], the prevalence rates of any hypoglycemia within the study period were 12.4%, 25.1%, and 32.2% for the ICD-9, NLP algorithm, and combined algorithm, respectively. Similarly, Misra-Hebert et al [35] found that NLP identified higher nonserious hypoglycemia events than ICD codes (14,763 vs 10,205 events) during the study period from 2005 to 2017; among 204,517 patients with no ICD codes for nonsevere hypoglycemia, evidence of nonsevere hypoglycemia was found in 7035 (3.44%) using NLP. Li et al [34] also showed that hypoglycemia was identified in 21% of the participants, with 9.67% identified only by NLP algorithms. In addition, Uzoigwe et al [36] found that the prevalence rates of hypoglycemia were 11.4% and <0.1% using NLP algorithms and ICD codes, respectively, in T2D; the prevalence rates were 20.4% and 0.1%, respectively, in T1D.

Using the combination of NLP and other approaches (eg, ICD codes) identified the highest prevalence or incidence of hypoglycemia compared with either method alone. Nunes et al [31] found that the prevalence rates of hypoglycemia were 12.4% for ICD codes, 25.1% for NLP algorithm, and 32.2% for combined algorithms; the incidence rates per 100 person-years were 2.3%, 4.8%, and 6.3% using ICD codes, NLP, and combined algorithms, respectively. Similarly, Misra-Hebert et al [35] identified that the incidence proportions of patients in the period from 2005 to 2017 were 0.4% and 1.3% for nonsevere hypoglycemia when using only ICD codes, whereas when NLP was added, the incidence proportions increased to 0.8% and 2.6%.

## Discussion

### Principal Findings

This systematic review aimed to synthesize the literature on the application of NLP to extract hypoglycemia from EHR clinical notes. Of the 8 studies, 4 (50%) reported that the prevalence rate of any level of hypoglycemia was 3.4% to 46.2%. Overall, the use of NLP to analyze clinical notes improved the capture of hypoglycemic events that may have been undocumented or missed using laboratory testing or ICD-9 and ICD-10 codes. The combination of NLP and other approaches significantly increased the identification of hypoglycemic events compared with individual methods. All reviewed studies applied rule-based NLP methods to identify hypoglycemia.

Previous reviews of the prevalence and incidence of hypoglycemia using NLP are limited. Our study found that the prevalence rate of any condition of hypoglycemia was 3.4% to 46.2%, whereas a previous review study reported that the prevalence rate of any condition of hypoglycemia ranged from 1% to 19% for studies using EHR as a data source [8]. In addition, 13% (1/8) of the studies in our review reported that symptom-based hypoglycemia—the estimated prevalence rate of hypoglycemia using combined symptom-based and nonsymptom-based definitions—was 20.4% (T1D) and 11.4% (T2D) [36], which is more prevalent than previous analyses without applying NLP for data extraction [40,41].

All included studies (n=8) applied rule-based NLP to identify hypoglycemia. The main aim of our paper focused on the application of NLP algorithms to identify hypoglycemia and not on the method for developing algorithms. Published articles have reported developing machine learning or deep learning algorithms to identify hypoglycemia, but they did not report the incidence of hypoglycemia; therefore, we did not include such papers in our review. For example, Chen et al [42] incorporated 3 machine learning algorithms to detect hypoglycemia, including logistic regression, linear support vector machines, and random forest. The result showed that single cross-validation logistic regression with cost-sensitive learning achieved the best performance with sensitivity of 0.693 and specificity of 0.974. In addition, Jin et al [43] developed and evaluated deep learning–based NLP systems to automatically detect hypoglycemia events from EHR narratives; they found that the convolutional neural network model yielded a promising performance with precision of 0.96 and recall of 0.86 in a 10-fold cross-validation setting. Furthermore, none of our reviewed studies applied the currently dominant method (eg, transformer models and transfer learning) in NLP research to identify hypoglycemia from EHR data. Our review indicated that the applications of NLP to identify hypoglycemia mainly use the rule-based system. Although machine learning– and deep learning–based algorithms have been developed, they have not been applied in clinical research.

A limitation of this review is the heterogeneity of the reported results. This heterogeneity prevents the estimation of the pooled incidence and prevalence of hypoglycemia in diabetes using NLP algorithms. In addition, excluding conference proceedings reduced the number of papers included. However, medical literature does not take conference proceedings into much consideration when making clinical decisions; therefore, conference proceedings are usually not included in a review paper in medical literature. However, in terms of clinical impacts, findings from the excluded conference proceedings would have more impact regarding the clinical decision of using NLP as a clinical algorithm, which can help patients or physicians to better identify high-risk hypoglycemia. To the best of our knowledge, this is the first systematic review to synthesize the prevalence and incidence of hypoglycemia using NLP in individuals with diabetes. All reviewed studies applied the combination of NLP with ICD codes and laboratory testing and identified higher incidence of hypoglycemia when using EHR data sources. This has significant clinical implications for the prevention and management of hypoglycemia; with the widespread use of EHRs, leveraging clinical notes significantly improves the identification of individuals with hypoglycemia. The preferred strategy is to use structured data (ICD codes), followed by using NLP to synthesize the unstructured data to pinpoint those at highest risk for hypoglycemia.

### Conclusions

In conclusion, our findings provided evidence that the application of NLP to analyze clinical notes improved the capture of hypoglycemic events, particularly when combined with ICD-9 and ICD-10 codes and laboratory testing. Identifying such patients with diabetes is important and necessary for characterizing treatment and unmet needs, thus preventing the adverse events and mortality associated with hypoglycemia. The current application of NLP in the identification of hypoglycemia still relies on the traditional rule-based methods; although machine learning– and deep learning–based algorithms have been developed, they have not been applied in clinical research. Future research should explore comparison of the rule-based systems, machine learning approaches, and deep learning–based NLP methods (eg, transformer models and transfer learning) to improve NLP efficiency.

## Abbreviations

EHR: electronic health record
HbA_1c_: glycated hemoglobin
ICD: International Classification of Diseases
ICD-10: International Classification of Diseases, Tenth Revision
ICD-9: International Classification of Diseases, Ninth Revision
NLP: natural language processing
PRISMA: Preferred Reporting Items for Systematic Reviews and Meta-Analyses
T1D: type 1 diabetes
T2D: type 2 diabetes
